# Structure of human saposin A at lysosomal pH

**DOI:** 10.1107/S2053230X15008584

**Published:** 2015-06-27

**Authors:** Chris H. Hill, Randy J. Read, Janet E. Deane

**Affiliations:** a Department of Haematology, Cambridge Institute for Medical Research, University of Cambridge, Wellcome Trust/MRC Building, Cambridge Biomedical Campus, Hills Road, Cambridge CB2 0XY, England

**Keywords:** saposin A, lipid-transfer protein, sphingolipid activator protein, GALC

## Abstract

The saposins are essential cofactors for the normal lysosomal degradation of complex glycosphingolipids by acid hydrolase enzymes; defects in either saposin or hydrolase function lead to severe metabolic diseases. Saposin A (SapA) activates the enzyme β-galactocerebrosidase (GALC), which catalyzes the breakdown of β-d-galactocerebroside, the principal lipid component of myelin. SapA is known to bind lipids and detergents in a pH-dependent manner; this is accompanied by a striking transition from a ‘closed’ to an ‘open’ conformation. However, previous structures were determined at non-lysosomal pH. This work describes a 1.8 Å resolution X-ray crystal structure determined at the physiologically relevant lysosomal pH 4.8. In the absence of lipid or detergent at pH 4.8, SapA is observeed to adopt a conformation closely resembling the previously determined ‘closed’ conformation, showing that pH alone is not sufficient for the transition to the ‘open’ conformation. Structural alignments reveal small conformational changes, highlighting regions of flexibility.

## Abstract 2

A 1.8 Å resolution structure of the sphingolipid activator protein saposin A has been determined at pH 4.8, the physiologically relevant lysosomal pH for hydrolase enzyme activation and lipid-transfer activity.

## 1. Introduction   

The lysosomal breakdown of complex glycosphingolipids (GSLs) is essential for normal cellular homeostasis and recycling of essential macromolecular building blocks. GSL substrates for degradation are embedded in membranes of lysosomal intralumenal vesicles, where complex glycan head groups are disassembled one residue at a time by the sequential action of lysosomal acid hydrolase enzymes (Sandhoff & Kolter, 1996 ▸; Kolter & Sandhoff, 2005 ▸, 2010 ▸). The active sites of these enzymes are typically shallow; in a membrane environment the steric crowding of head groups and lateral association of GSLs into clusters prevents enzymes from accessing the scissile bonds of their target substrates. Saposins A, B, C and D comprise a family of small, non-enzymatic lipid-transfer proteins that are essential for the activation of GSL catabolism; genetic defects in saposin function cause specific forms of lysosomal storage diseases (Spiegel *et al.*, 2005 ▸; Sun *et al.*, 2010 ▸; Sun & Grabowski, 2013 ▸). The four saposins are derived from proteolytic cleavage of prosaposin, which is expressed as a single gene product (Hiraiwa *et al.*, 1997 ▸). Each saposin shows functional specificity for the activation of certain catabolic reactions: saposin A (SapA) activates the hydrolysis of β-d-galactocerebroside to ceramide and galactose by the enzyme β-galactocerebrosidase (GALC; EC 3.2.1.46; Morimoto *et al.*, 1989 ▸; Harzer *et al.*, 1997 ▸).

The structures of all four saposins have previously been determined by X-ray crystallography (Ahn, Faull, Whitelegge, Fluharty *et al.*, 2003 ▸; Ahn *et al.*, 2006 ▸; Rossmann *et al.*, 2008 ▸; Popovic & Privé, 2008 ▸) or NMR spectroscopy (de Alba *et al.*, 2003 ▸). Whilst they share less than 35% sequence identity, they all adopt a common fold consisting of four amphipathic α-helices organized into two ‘fingers’. This fold forms the basis of a large, diverse superfamily of lipid-binding saposin-like proteins (SAPLIPs), members of which are found amongst phylogenetically distant eukaryotes, including metazoa, plants and mammals (Bruhn, 2005 ▸).

Previous structural work has described SapA in a monomeric ‘closed’ conformation in the absence of lipid (Ahn *et al.*, 2006 ▸) and more recently a homodimeric ‘open’ conformation in lipoprotein discs formed by the presence of the detergent lauryl­dimethylamine oxide (LDAO; Popovic *et al.*, 2012 ▸). These structures were determined at pH 6.0 and 6.5, respectively. However, SapA dimerization, lipid binding and membrane excavation only occurs at acidic pH (Vaccaro *et al.*, 1995 ▸; Ahn *et al.*, 2006 ▸; Locatelli-Hoops *et al.*, 2006 ▸) and it is unclear what role pH plays in the transition between the ‘closed’ and ‘open’ conformations. The aim of this study was to determine the structure of SapA at a lysosomal pH, thus generating an additional snapshot of the protein at a pH where it is functionally active, providing insights into pH-dependent conformational changes.

## 2. Materials and methods   

### 2.1. Cloning, protein expression and purification   

A codon-optimized, untagged cDNA encoding human SapA was synthesized by GeneArt and cloned into expression vector pET-15b using NcoI and XhoI restriction-endonuclease sites. The resultant construct was checked by sequencing with the T7F primer (5′-TAATACGACTCACTATAGGG-3′). The amino-acid sequence of this construct (MGSLPCDICKDV­VTAAGDMLKDNATEEEILVYLEKTCDWLPKPNMSASCKEIVDSYLPVILDIIKGEMSRPGEVCSALNLCESLQ) is equivalent to residues 60–142 of the precursor prosaposin (UniProt entry P07602), with an additional Met-Gly at the N-terminus as a result of the cloning strategy.

The protein was expressed in *Escherichia coli* Origami (DE3) cells and cultures (1 l) were grown in 2×TY medium supplemented with 50 µg ml^−1^ ampicillin and 12.5 µg ml^−1^ tetracycline (37°C with shaking at 230 rev min^−1^). Expression was induced for 4 h at 37°C by the addition of 1.0 m*M* isopropyl β-d-1-thiogalactopyranoside (IPTG) when the culture reached an optical density at 600 nm of 0.6. The cells were harvested by centrifugation (5000*g*, 20 min) and washed with phosphate-buffered saline prior to storage at −20°C.

The presence of three disulfide bonds per saposin molecule confers remarkable thermal stability, which has previously been exploited for protein purification without affinity tags (Ahn, Faull, Whitelegge, Higginson *et al.*, 2003 ▸; Ahn *et al.*, 2006 ▸). Bacterial pellets from 1 l culture were thawed and resuspended in 30 ml ice-cold anion-exchange (AEX) buffer (50 m*M* Tris pH 7.4, 25 m*M* NaCl). This was passed twice through a hydraulic cell disruptor (Constant Systems TS) at 206 MPa pressure and 8°C. Insoluble cell-wall and membrane fractions were pelleted by centrifugation (40 000*g*, 40 min, 4°C) and discarded. The cleared lysate was then heat-treated in a water bath (100°C, 30 min). Denatured, precipitated *E. coli* native proteins were then pelleted by centrifugation (40 000*g*, 40 min, 4°C) and discarded. The supernatant was then supplemented with 20 µg ml^−1^ DNase I, 2.5 m*M* MgCl_2_, 0.5 m*M* CaCl_2_ and incubated with gentle mixing (room temperature, 4 h). This crude 50 ml extract was then dialyzed overnight (3500 MWCO, Thermo Scientific) against 5 l AEX buffer.

A 5.0 ml HiTrap QSepharose column (GE) was equilibrated with 10 column volumes (CV) of AEX buffer before loading the crude SapA extract using a peristaltic pump at 5.0 ml min^−1^. The column was then washed with 10 CV AEX buffer and connected to an FPLC (ÄKTApurifier, GE) for elution of bound proteins with a 0–50% gradient of 50 m*M* Tris pH 7.4, 1 *M* NaCl over 20 CV. All fractions containing saposins were pooled and concentrated to a volume of 5.0 ml using Vivaspin 20 centrifugal concentrators (3000 MWCO, GE). A HiLoad 16/600 Superdex 75 column (GE) connected to the FPLC was equilibrated with 1.5 CV size-exclusion (SEC) buffer (50 m*M* Tris pH 7.4, 150 m*M* NaCl) prior to the injection of 5.0 ml of the concentrated AEX elution. Isocratic elution with SEC buffer was performed at 1.0 ml min^−1^ over 1.2 CV. Fractions corresponding to the main *A*_280_ peak were pooled and stored at 4°C. Purified SapA was stable for at least six months.

### 2.2. Crystallization   

**Figure 1:**
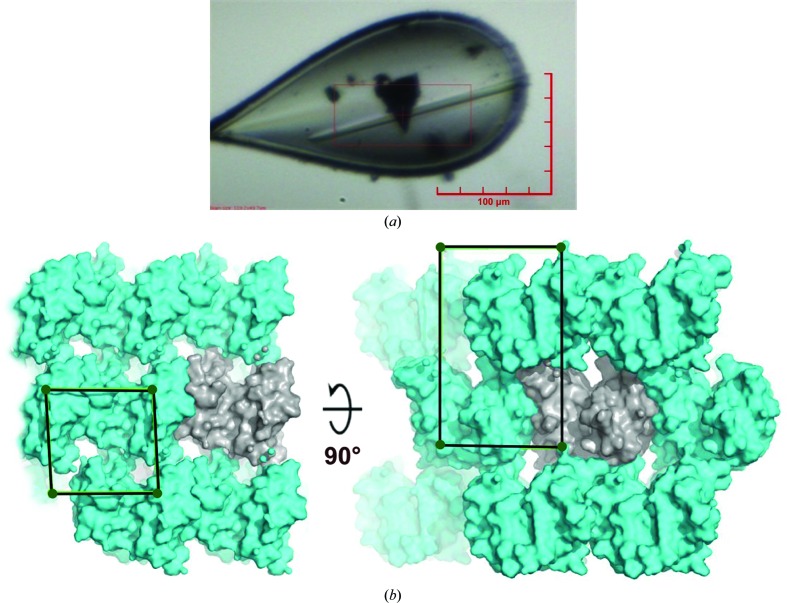
Human SapA crystallization and mounting. (*a*) SapA crystalline needle cluster grown by sitting-drop vapour diffusion against a reservoir consisting of 0.1 *M* sodium acetate pH 4.8, 0.2 *M* lithium sulfate, 25%(*w*/*v*) PEG 4000. A single needle (10 × 150 µm) is shown mounted in a nylon loop. (*b*) Details of crystal packing, showing views of the unit cell along the *b* (left) and *a* (right) axes. Two protein molecules are present per asymmetric unit (grey). The crystals contain 33.2% solvent, with a Matthews coefficient of 1.84 Å^3^ Da^−1^.

Human SapA was concentrated to 19.6 mg ml^−1^ in 150 m*M* NaCl, 50 m*M* Tris pH 7.4 using Amicon Ultra 3.5 kDa molecular-mass centrifugal concentrators (Millipore). Crystallization experiments were set up in 96-well sitting-drop vapour-diffusion plates using an Innovadyne Screenmaker 96+8 Xtal microfluidic handling platform. Diffraction-quality crystals were grown in 400 nl drops (200 nl protein and 200 nl reservoir solution) at 293 K by equilibration against an 80 µl reservoir consisting of 0.2 *M* lithium sulfate, 0.1 *M* sodium acetate pH 4.8, 25%(*w*/*v*) polyethylene glycol 4000. The final pH of the crystallization drop was verified experimentally as pH 4.8. Crystals grew as clusters of needles and were dissected with an acupuncture needle before mounting to obtain single crystals (Fig. 1 ▸ *a*), which were cryoprotected with 20%(*v*/*v*) glycerol and flash-cooled in liquid nitrogen.

### 2.3. Data collection and processing   

Data-collection and processing statistics are detailed in Table 1 ▸. Crystals were maintained at a temperature of 100 K during data collection. Diffraction data were recorded on beamline I03 at Diamond Light Source using a Pilatus 6M detector (Dectris). Data sets of 1800 images were collected at λ = 0.9763 Å over a 360° oscillation range with Δϕ = 0.2°, *t* = 0.2 s, transmission = 18.05% (∼3.07 × 10^11^ photons s^−1^). Diffraction data were processed using the *xia*2 pipeline (Winter, 2010 ▸) implementing *XDS* (Kabsch, 2010 ▸) for data indexing and integration, *POINTLESS* (Evans, 2006 ▸) for point-group determination and *SCALA* (Evans, 2006 ▸) for scaling and merging. The resolution cutoff was decided by CC_1/2_ > 0.5 in the outer resolution shell (Karplus & Diederichs, 2012 ▸).

### 2.4. Structure solution, refinement and analysis   

The structure was solved by molecular replacement with *Phaser* (McCoy *et al.*, 2007 ▸), using the known structure of human SapA at pH 6.0 (PDB entry 2dob; Ahn *et al.*, 2006 ▸) as a search model. All further model adjustment and refinement was performed iteratively using *Coot* (Emsley *et al.*, 2010 ▸) and *phenix.refine* (Afonine *et al.*, 2012 ▸) (Table 2 ▸). H atoms were refined in the riding position. Validation tests implemented in *MolProbity* (Chen *et al.*, 2010 ▸) were consulted throughout the refinement process. For the electrostatic potential calculations, partial charges were assigned using the *PDB*2*PQR* server (Dolinsky *et al.*, 2004 ▸), implementing *PROPKA* (Li *et al.*, 2005 ▸) to estimate side-chain p*K*_a_ values. Electrostatic surfaces were calculated using *APBS* (Baker *et al.*, 2001 ▸) and structural figures were rendered using *PyMOL* (Schrödinger). Sequence-alignment graphics were prepared using *ALINE* (Bond & Schüttelkopf, 2009 ▸). The atomic coordinates and structure factors have been deposited in the PDB under accession code 4uex.

## 3. Results and discussion   

### 3.1. SapA remains in the ‘closed’ conformation at lysosomal pH 4.8   

**Figure 2:**
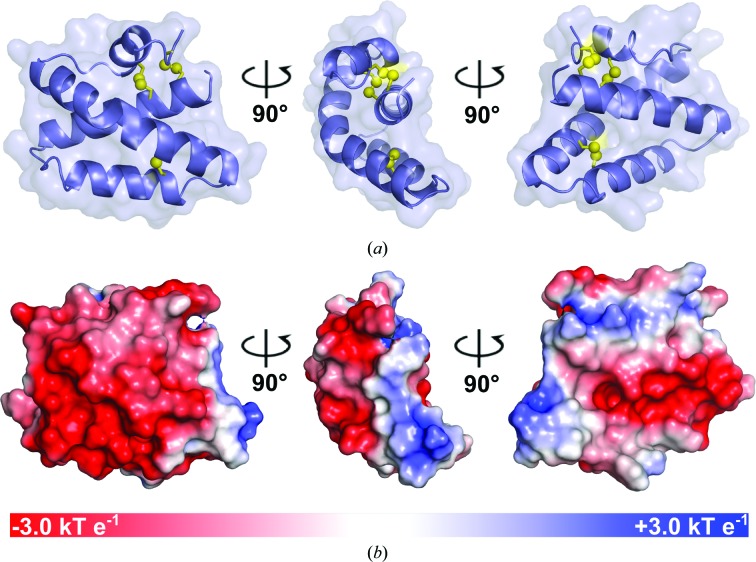
X-ray crystal structure of SapA at lysosomal pH. (*a*) Monomeric SapA is shown in three orientations. Disulfide bonds are shown as yellow spheres. (*b*) The molecular surface is shown coloured by electrostatic potential at the solvent-accessible surface from red (negative, −3.0 *kT* e^−1^) to blue (positive, +3.0 *kT* e^−1^). Electrostatic potential was calculated using a pH value of 4.8 when assigning side-chain protonation.

Human SapA crystals belong to the monoclinic space group *P*2_1_ and have a relatively low solvent content of 33.2% as estimated by the Matthews method (Matthews, 1968 ▸; *V*_M_ = 1.84 Å^3^ Da^−1^; Fig. 1 ▸ *b*). The asymmetric unit contains two molecules of SapA, both in the closed conformation, with the characteristic saposin fold (Ahn, Faull, Whitelegge, Fluharty *et al.*, 2003 ▸; Ahn *et al.*, 2006 ▸). Each monomer consists of two ‘fingers’ composed of an α1–α4 stem and an α2–α3 hairpin (Fig. 2 ▸ *a*). These motifs are secured by three intramolecular disulfide bridges connecting the terminal helices α1 and α4 and the central helices α2 and α3. Helix α3 is kinked at the conserved residue Tyr54. The helices are amphipathic and are oriented such that hydrophobic lipid-binding side chains are buried in the centre of the ‘closed’ conformation. Acidic residues comprise the solvent-accessible outer surface, creating an estimated electrostatic surface potential of −8.0 e at lysosomal pH 4.8, although small basic and neutral patches remain (Fig. 2 ▸ *b*). As the theoretical isoelectric point of SapA is ∼4.2, the charge distribution seen here is not significantly different from that at pH 6.0–7.4. Two flexible coil regions act as hinges, allowing movement of the ‘fingers’ relative to one another to accommodate lipid or detergent molecules, as previously shown for saposins A, B and C (Ahn, Faull, Whitelegge, Fluharty *et al.*, 2003 ▸; Hawkins *et al.*, 2005 ▸; Ahn *et al.*, 2006 ▸; Popovic *et al.*, 2012 ▸). In the case of SapA, upon transition to the open conformation, the α3 helix is extended by the coil residues 63–67 and the kink at Tyr54 is preserved.

### 3.2. Crystal packing is pseudosymmetric   

In this structure, two molecules were observed in the asymmetric unit. The two molecules are related by an approximate 2_1_ screw axis parallel to the *c* cell edge. With the unit-cell β angle of 93.1°, the overall symmetry deviates substantially from space group *P*2_1_2_1_2_1_. Analysis with *PDBePISA* (Krissinel & Henrick, 2007 ▸) suggests that the two molecules in the asymmetric unit form a crystallographic pseudodimer and not a stable quaternary complex. This is consistent with observations that SapA remains monomeric at low pH in the absence of detergent (Ahn *et al.*, 2006 ▸).

### 3.3. Structural alignment reveals regions of flexibility   

**Figure 3:**
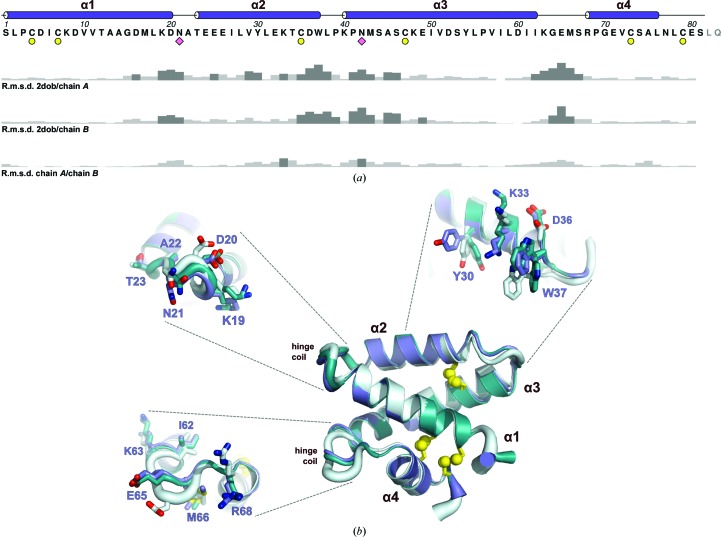
Alignment of monomeric, closed SapA structures. (*a*) Sequence diagram of human SapA. The highlighted features are cysteines that form disulfide bonds (yellow circles) and asparagines where N-linked glycosylation occurs (pink diamonds). The bar graphs beneath the sequence show the backbone r.m.s.d. per residue after least-squares fit alignment of PDB entry 2dob with chain *A*, of PDB entry 2dob with chain *B* and of chain *A* with chain *B*. Dark grey bars indicate r.m.s.d > 1.0 Å. (*b*) Least-squares fit alignment of the human SapA structure determined here (chain *A*, purple; chain *B*, teal) with the previous structure at pH 6.0 (PDB entry 2dob, grey). Ribbon thickness in nonhelical regions is proportional to atomic *B* factor. Details of the greatest conformational differences are shown as sticks, including flexible hinge coil regions (indicated) and the outer surface of the α2 helix.

The two chains observed in the asymmetric unit are almost identical, with a backbone r.m.s.d. of 0.46 Å after least-squares alignment. Inspection of side-chain dihedral angles revealed small conformational changes in a few residues as a result of crystal packing, including Lys19, Glu24, Glu25, Lys33, Ser46, Ile50 and Glu65. The largest difference was seen at Trp37, which was observed in a dual-occupancy conformation in chain *B* only, indicating that this side chain is mobile. Alignment of both chains in this structure with the SapA structure at pH 6.0 (Ahn *et al.*, 2006 ▸; PDB entry 2dob) reveals additional conformational changes, with backbone r.m.s.d. values of 0.94 Å for both chains after least-squares fit alignment (Fig. 3 ▸ *a*). The largest differences are observed at the ends of helices and in the flexible hinge regions; residues 19–21 between α1 and α2 and residues 62–67 between α3 and α4.

Interestingly, several residues on the solvent-accessible face of helix α2 are observed in different conformations in all three chains, indicating these side chains to be highly mobile. *In vitro* studies of the interaction of SapA with phosphatidylserine (PS) liposomes using tryptophan fluorescence spectroscopy and quenching experiments showed that the Trp37 side chain can associate with lipids and is thought to insert into membranes by about five carbon-bond lengths (Qi & Grabowski, 2001 ▸). Similarly, measurements of tryptophan fluorescence within SapA lipoprotein discs show this residue to be lipid-associated, regardless of lipid composition (Popovic *et al.*, 2012 ▸). The conformational flexibility observed in this region could facilitate the association of closed, monomeric SapA with membranes prior to lipid binding and lipoprotein disc formation *via* membrane insertion of the bulky hydrophobic residues Tyr30 and Trp37. Residue Tyr30 is very well conserved amongst vertebrate SapA orthologues, and Trp37, although not fully conserved, is substituted by other hydrophobic residues (phenylalanine or leucine), supporting an important functional role for these residues. Interestingly, Tyr30 and Trp37 are not conserved across the different saposin molecules, suggesting that the role of these residues in lipid interactions differs between saposins.

In contrast, helices α1, α3 and α4 have low backbone r.m.s.d. values and mostly identical side-chain conformations (Fig. 3 ▸ *b*). Comparison of atomic *B* factors shows that the α3–α4 hinge region is more ordered in both chains of the pH 4.8 structure than in the pH 6.0 structure. However, owing to the low solvent content and pseudosymmetry observed in these crystals, it is difficult to determine whether the differences observed here are a result of pH alone or are influenced by crystal packing.

## 4. Conclusions   

There are no large-scale conformational rearrangements accompanying pH change, emphasizing the absolute necessity of lipids or detergents for transition to the open conformation (Ahn *et al.*, 2006 ▸; Popovic *et al.*, 2012 ▸). However, in combination with the structure of closed SapA at pH 6.0, this work reveals regions of conformational flexibility, and the crystal packing suggests a tendency to bury the negatively charged α3 helix at lysosomal pH. This may have implications for a direct interaction with the hydrolase enzyme GALC, which has a positive surface charge surrounding the active site at lysosomal pH (Deane *et al.*, 2011 ▸; Hill *et al.*, 2013 ▸).

## Supplementary Material

PDB reference: saposin A, 4uex

## Figures and Tables

**Table 1:** Data collection and processing Values in parentheses are for the outer shell.

Space group	*P*2_1_
*a*, *b*, *c* ()	34.17, 58.69, 35.16
, , ()	90.0, 93.1, 90.0
Mosaic spread ()	0.146
Resolution range ()	34.121.80 (1.861.80)
Completeness (%)	98.6 (97.7)
Multiplicity	6.8 (6.8)
*I*/(*I*)†	11.2 (1.8)
CC_1/2_ ‡	0.997 (0.676)
*R*_meas_	0.122 (1.182)
Overall *B* factor from Wilson plot (^2^)	22.72

† Mean *I*/(*I*) is >2.0 at resolutions of >1.9.

‡ The CC_1/2_ values were used to decide the resolution cutoff (Karplus Diederichs, 2012 ▸).

**Table 2:** Structure solution and refinement Values in parentheses are for the outer shell.

Resolution range ()	34.121.80 (1.861.80)
No. of reflections, working set	12126 (1179)
No. of reflections, test set	612 (64)
Final *R*_work_	0.186
Final *R*_free_	0.210
No. of non-H atoms	
Protein	1304
Water	26
Total	1330
R.m.s. deviations	
Bonds ()	0.004
Angles ()	0.807
Average *B* factors (^2^)	
Protein	25.4
Water	31.6
Ramachandran plot	
Most favoured (%)	98.8
Allowed (%)	1.2
Outliers (%)	0
